# Supplementary material for: Spatial Variation in Stream-Water Bacterial Communities Across Sites Differing in Wild Chinese Giant Salamander Detection Frequency
Source: Biology (Basel). 2026 Sep 18;15(18):1647. doi: 10.3390/biology15181647 (PMC13604818; doi:10.3390/biology15181647)
Supplement: Supplementary file 1 [file biology-15-01647-s001.zip › Data Related to “Spatial Variation in Stream-Water Bacterial Communities across Sites Differing in Wild Chinese Giant Salamander Detection Frequency”/Microbial Data/result/08.FunctionPrediction/picrust2/heatmap/sample/heatmap.sample.EC.cluster.pdf]

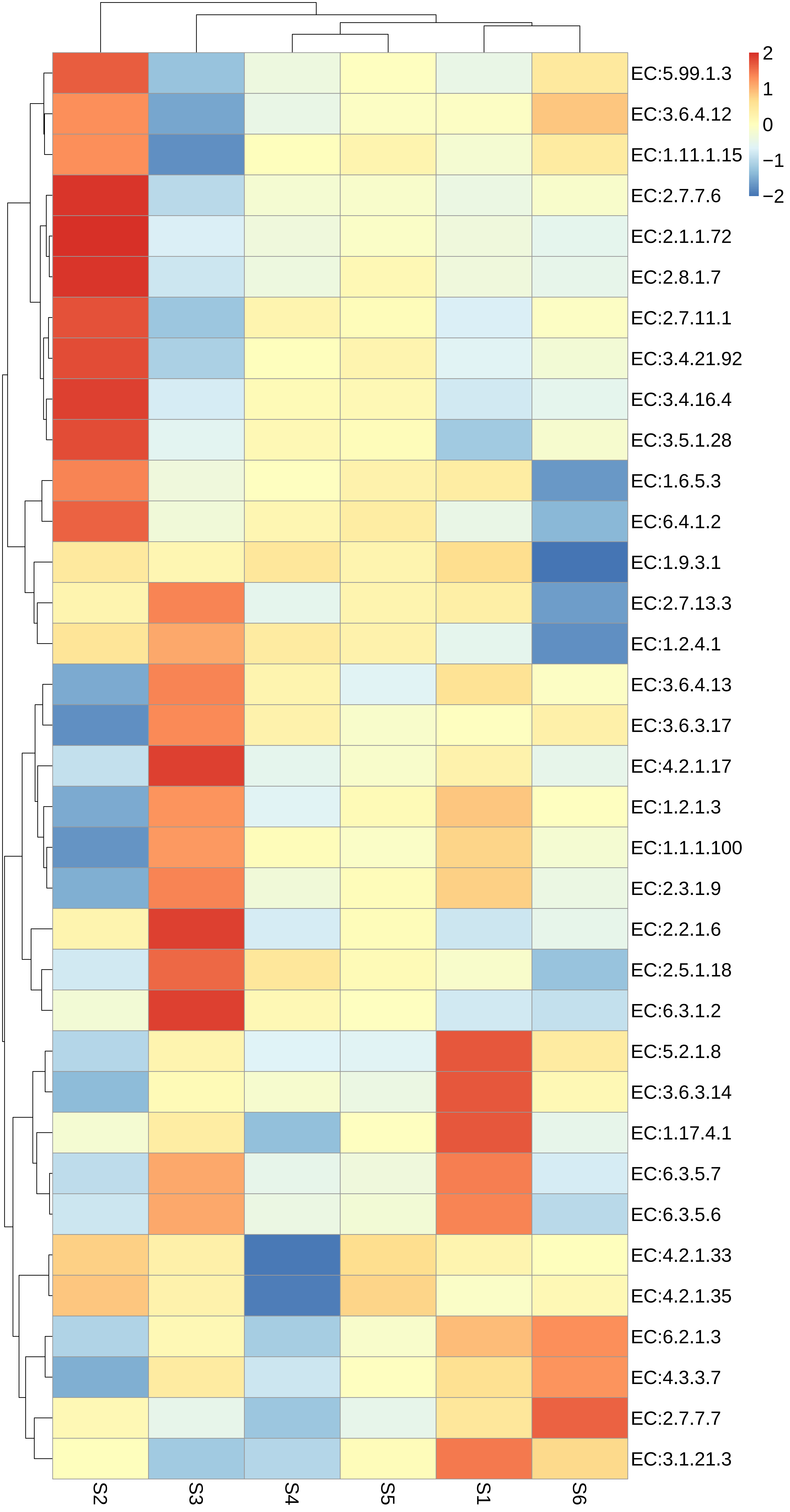

2
1
0
−1
−2
EC:5.99.1.3
EC:3.6.4.12
EC:1.11.1.15
EC:2.7.7.6
EC:2.1.1.72
EC:2.8.1.7
EC:2.7.11.1
EC:3.4.21.92
EC:3.4.16.4
EC:3.5.1.28
EC:1.6.5.3
EC:6.4.1.2
EC:1.9.3.1
EC:2.7.13.3
EC:1.2.4.1
EC:3.6.4.13
EC:3.6.3.17
EC:4.2.1.17
EC:1.2.1.3
EC:1.1.1.100
EC:2.3.1.9
EC:2.2.1.6
EC:2.5.1.18
EC:6.3.1.2
EC:5.2.1.8
EC:3.6.3.14
EC:1.17.4.1
EC:6.3.5.7
EC:6.3.5.6
EC:4.2.1.33
EC:4.2.1.35
EC:6.2.1.3
EC:4.3.3.7
EC:2.7.7.7
EC:3.1.21.3
S2
S3
S4
S5
S1
S6
